# Using white noise to gate organic transistors for dynamic monitoring of cultured cell layers

**DOI:** 10.1038/srep11613

**Published:** 2015-06-26

**Authors:** Jonathan Rivnay, Pierre Leleux, Adel Hama, Marc Ramuz, Miriam Huerta, George G. Malliaras, Roisin M. Owens

**Affiliations:** 1 Department of Bioelectronics, Ecole Nationale Superieure des Mines, CMP-EMSE, MOC, 13541 Gardanne, France; 2 Microvitae Technologies, Hôtel Technologique, Europarc Sainte Victoire Bât 6 Route de Valbrillant, 13590 Meyreuil, France

## Abstract

Impedance sensing of biological systems allows for monitoring of cell and tissue properties, including cell-substrate attachment, layer confluence, and the “tightness” of an epithelial tissue. These properties are critical for electrical detection of tissue health and viability in applications such as toxicological screening. Organic transistors based on conducting polymers offer a promising route to efficiently transduce ionic currents to attain high quality impedance spectra, but collection of complete impedance spectra can be time consuming (minutes). By applying uniform white noise at the gate of an organic electrochemical transistor (OECT), and measuring the resulting current noise, we are able to dynamically monitor the impedance and thus integrity of cultured epithelial monolayers. We show that noise sourcing can be used to track rapid monolayer disruption due to compounds which interfere with dynamic polymerization events crucial for maintaining cytoskeletal integrity, and to resolve sub-second alterations to the monolayer integrity.

Cells are reactive to process stimuli they receive from the environment, and the speed of the response is known to depend not only on the mechanism of delivery of the signal but also on the nature of the response^1^. In the case where the response involves interactions with already formed proteins, the signal processing is considered ‘fast’ (msec-sec)^2^. The more intermediate steps involved, however, the longer the process. In the case where the extracellular signal relies on transcription and translation of new protein for the effect to occur, these signaling processes are considered ‘slow’ (multiple minutes to hours). The process by which neurotransmitters cause an allosteric change in the conformation of a gated ion channel embedded in the cell membrane is, for example, a very rapid (msec) process, as it requires diffusion of a molecule across a narrow synaptic cleft and then binding to the membrane receptor in the post-synaptic cell^2^. A slightly slower, although still fast process (seconds), is the case of protein phosphorylation, where the addition of a phosphate group via a kinase, causes a change in protein function in the cell^3^. Actin cystoskeletal rearrangements represent an example of another rapid process; Actin is a cytoskeletal protein necessary for a wide variety of cellular processes including cell migration, adhesion, polarization, and more. The cytoplasm of the cell contains a pool of actin subunits which can rapidly polymerize to form filaments necessary to carry out the aforementioned processes, on timescales of seconds to a few minutes^4^. Actin dynamics are mediated via a vast array of accessory proteins and triggered by numerous different signaling pathways, including protein phosphorylation pathways^5^.

Traditionally, cell biologists have relied on optical methods for determining the effects of extracellular stimuli. In many cases these methods are static, involving fixed cells; increasingly, dynamic live cell imaging techniques such as FRAP (fluorescence recovery after photobleaching), and FRET (fluorescence resonance energy transfer) may be used. In terms of time resolution, the limiting factor is usually exposure time required by the experiment/camera which may be in the millisecond range for more routine techniques, but in the second range for newer techniques such as PALM (photo-activated localization microscopy) or STORM (stochastical optical reconstruction microscopy) which attempt to improve the *spatial* resolution into the low nanometer range^6^. Electrical methods for monitoring cell response to stimuli are emerging as viable alternatives, in particular where the methods are label-free and continuous, and not limited by the diffraction limit in contrast to most optical methods^7^. This is particularly interesting for monitoring of cellular responses to stimuli that may occur on the msec or sec time regime, where important information may be lost due to limitations in the measurement techniques. One drawback for electrical monitoring techniques is the inability to unambiguously correlate the electrical signal with an event occurring in the cell, however the use of combined optical and electronic monitoring has the potential to resolve this issue^8^. Cell-based impedance studies provide important information about the nature and ‘integrity’ of cells on or near an electrode surface and have provided insight into cell adhesion, cell-substrate distance, barrier tissue properties, and micromotion of cells^7^,^9^,^10^,^11^,^12^,^13^,^14^.

Typical impedance analysis for toxicological monitoring of live cells is performed by sweeping frequency, as with a traditional potentiostat^9^,^15^, or by applying more complex compound waveforms, such as multiple sinusoidal inputs superimposed, or a chirp for example. Applying a signal which instantaneously contains a broad frequency range is advantageous for dynamic monitoring. In this case, a pulse, step, or white noise can be most beneficial. Such approaches, especially the input of noise, is prominently used to monitor the impulse response of an electrical circuit (for example, amplifiers), or in testing audio equipment or acoustics in a concert hall. Noise has been used to interrogate biological systems on a limited basis^16^. The ‘color’ of noise, in the present case, white, implies a flat (constant) signal content over a broad frequency range. Applying white noise is analogous to white light spectroscopy, where the input light source contains a broad spectrum of (visible) wavelengths, and can allow one to perform optical spectroscopy instantaneously given the proper detector and analysis routine.

Organic electronic materials are ideal candidates for interfacing with cells and biological tissues. Specifically, materials such as conducting polymers (CP) are soft, allowing for better mechanical matching with cells, can easily uptake water and exchange ions from the biological milieu, and are readily processable^17^. While CPs have been used as electrode coatings for lowered impedance and thus higher signal to noise ratio, their use as active materials in transistors provides added value. For sensing applications, the CP poly(3,4-ethylenedioxythiophene) doped with the polyanion poly(styrene sulfonate), or PEDOT:PSS, is commonly used as the channel material in organic electrochemical transistors (OECTs)^18^. In such a device, the PEDOT:PSS channel is submerged in an aqueous electrolyte, and is electrochemically doped or de-doped by an applied gate bias, or by a biologically sourced event which effectively gates the channel. The OECT has been shown to efficiently transduce ionic currents into electronic currents, through its high capacitance, leading to high transconductance^19^. As such the OECT has been used in a wide variety of biomedical applications^18^. Recently, our group demonstrated the ability to combine measurements of gate and drain current of an OECT to assemble broad-band impedance spectra of cultured epithelial monolayers^20^. Such an approach however, relies on harmonic frequency sweeps common to most EIS implementations. Thus, while the OECT in this case presents a distinct advantage in sensitively measuring ionic currents (ionic impedance), it is not readily translatable to very rapid dynamic monitoring applications addressed above.

In this work we employ uniform white noise as the gate voltage input of an OECT to achieve rapid monitoring at the subsecond to second resolution. By simultaneously monitoring the resulting gate and drain current noise, we are able to extract impedance of cultured epithelial monolayers in real time. We show the utility of such a technique for real time monitoring of the toxicological effects of a fungal toxin (Cytochalasin B), known to rapidly and profoundly affect the cell cytoskeleton^21^,^22^, and show impedance monitoring of the cellular response to this toxin with unprecedented temporal resolution. The data collection and analysis described herein allows for detection of early, fast (subsecond), and potentially subtle biological changes/disruption in response to internal/external stresses or toxic compounds.

## Results

**Figure 1:**
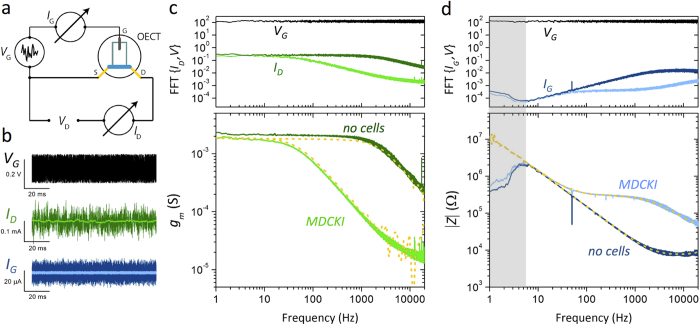
Noise-based impedance sensing with OECTs. **a.** Wiring diagram of an OECT for noise based sensing. *V*_*D*_ = −0.6 V, applied *V*_*G*_ is uniform white noise of amplitude 100 mV, *I*_*G*_ and *I*_*D*_ can be measured individually or simultaneously. **b.** Raw noise recording of applied white noise, *V*_*G*_ (black), as well as the measured current noise (*I*_*D*_, green; *I*_*G*_, blue) with cells (light colors) and without (dark colors). The FFT of 2 min noise recordings for the *I*_*D*_ based measurements (**c.**) yielding the transconductance vs. frequency response of the ionic circuit, and of the *I*_*G*_ based measurements (**d.**), providing an estimate of the system impedance. Dotted yellow lines are measurements of the same systems through a harmonic frequency sweeping approach. The grey region in (d.) is dominated by ambient current noise, rendering data in this region useless for impedance sensing using the gate current.

The OECT is wired as shown in Fig. 1a, measuring both drain and gate current simultaneously, with *V*_*D*_ = −0.6 V [Ref. 20]. The geometry (50 × 50 μm^2^, ~130 nm thick) of the OECT is selected such that the maximum transconductance (*g*_*m*_ = ∂*I*_*D*_/∂*V*_*G*_) is attained at *V*_*G*_ = 0 V, so that the applied gate noise requires no offset, and excessive biasing across the cell monolayer can be minimized^23^. A Ag/AgCl gate electrode is immersed in cell culture media, the electrolyte in this case, and uniform white noise of 100 mV amplitude is applied while the resulting current noise is measured (Fig. 1b). The data analysis relies on the fast Fourier transform (FFT) of the resulting signals; shown in Fig. 1c,d as the FFT of a 2 min recording, smoothed over 50 pts. The measurement of the applied white noise confirms the uniform contributions of signal at all frequencies in the 1 Hz–20 kHz range. The ratios of the absolute value of the measured current and voltage FFTs provide the frequency dependent transconductance, *g*_*m*_ = |FFT(*I*_*D*,noise_)|/|FFT(*V*_*G*,noise_)|, and impedance, |*Z*| = |FFT(*V*_*G*,noise_)|/|FFT(*I*_*G*,noise_)|. The shape and magnitude of the transconductance and impedance are in good agreement with results from a frequency scan experiment. In the latter case, a sinusoidal signal is applied at the gate, and the ratios of the input and output sine wave amplitudes yield the dotted yellow traces in Fig. 1. The deviation in |*Z*| of the noise results from the harmonic results at low frequencies is attributed to ambient noise in the measurement of low frequency gate current. The applied noise amplitude is selected carefully to minimize the exposure of excessive bias to the cells, but also to allow for proper recording of resulting device currents above the ambient background noise. An example of the transconductance dependence on *V*_*G*_ noise amplitude is shown in Supplementary Figure S1, where the drain current is almost entirely dominated by ambient noise when the applied noise is <~1 mV. These results suggest lower applied bias can be utilized for a more restricted frequency range, in environments with lower ambient noise, or with a transistor of higher transconductance. The effect of shorter duration recordings is also included as a reference (Supplementary Figure S2); as expected, the minimum duration of a noise recording depends on the frequency range of interest.

Immediate comparisons between a device without cells, and with cultured cells can be made due to the expected changes to the impedance^20^. Madin-Darby canine kidney epithelial cells (MDCK I) were chosen as a model cell layer due to their ability to readily form confluent cell layers with cell-cell junctions which limit permeability through the cell layer, and subsequently show large transepithelial electrical resistance (TER) values as measured with commercial impedance analysis systems such as the cellZscope (nanoAnalytics)^20^. The drain current and resulting transconductance show a lower cut-off frequency for the device with MDCK I compared to the device alone (Fig. 1c). This is due to the higher impedance of the cell monolayer, limiting the ionic current between the gate and channel of the device. This is immediately observable in the raw data (Fig. 1b, green), where the trace with cells appears to be low pass filtered–eliminating high frequency noise–due to the presence of the cells. The difference in impedance shows the device with cells has a plateau in the 100 Hz–1 kHz frequency range, which has been characterized previously using harmonic analysis^20^.

Since white noise is used as the input waveform, continuously applied noise can be used to dynamically monitor the impedance properties of a cell layer or tissue. Our previous work has relied on measurements every 2–10 minutes applying a pulse^24^,^25^, or performing a harmonic frequency sweep^20^. Here we monitor the cell layer integrity over the course of a 45 min experiment (performing harmonic sweeps before and after a 14 min noise measurement). After culturing MDCK I cells for 3 days on an OECT array, the transconductance and impedance are measured with a harmonic sweep. As a proof of principle of our system, we chose to monitor impedance related to changes in resistance of an epithelial cell layer after exposure to the fungal toxin Cytochalasin B (Cyt B). Cytochalasins are well-known inhibitors of actin polymerization, causing rapid and profound effects on the cytoskeleton of the cell. The cytochalasins are membrane permeable fungal compounds that bind tightly to one end of actin filaments thus inhibiting polymerization^26^. In epithelial cells, actin is present in microvilli and in the basal cytoplasm, but also found in a peripheral ring that surrounds the apical (top) part of the cell associated with the adherens junction (AD) and tight junctions (TJ) that facilitate cell-cell contacts^21^. TJs play a major role in regulating the permeability of epithelial layers to ions and other molecules and are associated with actin that exchanges very rapidly. FRAP monitoring of actin specifically at cell contacts was used to quantitate this exchange, and a mobile fraction of 98% was reported with t_1/2_ of 15–106 s depending on the number of days of the cells in culture^22^. Actin is known to play a significant role in controlling TJ permeability, and rapid decreases in TER (resistance of the epithelium to ion flow across the layer between adjacent cells) have been reported after treatment with Cyt B. A very high mobility and fast turnover of actin at cell contacts has been reported, due to rapid exchange with a large pool of cytoplasmic actin^27^. However, these reports may not reflect the actual kinetics due to limitations of the measurement technique.

**Figure 2:**
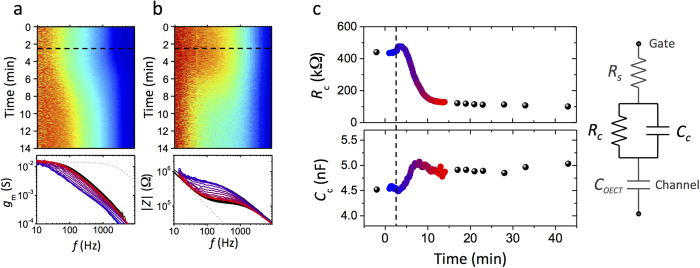
Application of noise-based impedance sensing to toxicology. **a.** Time-frequency spectrogram of transconductance (top; red high, blue low), and snapshots of transconductance vs. frequency upon addition of 8 μM Cytochalasin B (at t = 2.5 min, dashed lines) to media of MDCK I cells cultured on an OECT. **b.** Time-frequency analysis (red high, blue low) and snapshots of the simultaneously acquired impedance data (|*Z*|). The snapshots at time t = 0 min are blue, at t = 14 min are red. Black traces are from harmonic frequency sweep measurements before and after the continuous noise experiment. The dotted grey lines are data from the device alone (no cells). **c.** Cell layer resistance and capacitance of impedance data stitched from drain current and gate current recordings from a single experiment, and fit to the equivalent circuit shown on the right. Black symbols are from harmonic frequency sweep data, while blue-red data are from the noise results in (a,b).

We began the noise experiment (t = 0 min), and added 8 μM of Cytochalasin B at t = 2.5 min. The transconductance and impedance can be visualized using a time-frequency spectrogram (Fig. 2a,b), or by averaging over short time windows (in this case ~10 s) to extract *g*_*m*_ and |*Z*| curves like those in Fig. 1c,d. At such concentrations of toxin, a readily observable change in the barrier properties is measured over a 4 min duration. By comparison, a typical frequency sweep takes 1–2 min, which would limit the entire measurement of affected barrier function to only a few points that are likely skewed by rapid changes during the sweep of applied frequencies. The results in Fig. 2 are from a single representative noise experiment, which was performed multiple times (Supplementary Figure S3) showing similar dynamics and magnitudes of variation. The control experiments, where addition of cell culture media and DMSO (the Cytochalasin B solvent) are dynamically monitored using noise, are included as Supplementary Figure S4).

The resulting data is open to impedance analysis using a number of appropriate models. Here we employ a model as described previously^20^,^28^, with the equivalent circuit shown in Fig. 2, to fit the stitched impedance data and extract a resistance and capacitance of the cell layer (*R*_*c*_, *C*_*c*_). The capacitance of the OECT channel is first determined from an impedance fit of the device alone, giving *C*_*OECT*_ = 14.9 nF. This value is then fixed, while *R*_*c*_*, C*_*c*_ are kept free. A series resistance *R*_*s*_ is kept free: this term accounts for the media resistance in the case with no cells, and a sum of the media resistance and a term related to effective “cleft” resistance since the cells are cultured directly on top of the device. It should be noted that a specific cell area is not assumed, unlike the case with cell layers cultured on well-defined porous membranes, the effective cell layer area is not easily quantifiable. Nevertheless, the relative changes in the impedance can be quantified over the course of the experiment (Fig. 2c). The cell monolayer resistance (*R*_*c*_) decreases from ~425 kΩ to 100 kΩ, while the cell capacitance (*C*_*c*_) changes only slightly from 4.5 to 5 nF. *R*_*c*_ is thus the dominant term in the impedance variation, decreasing by 400–500 kΩ in multiple experiments; similarly, the magnitude and variation of *C*_*c*_ is also consistent upon repetition. The continuous change in *R*_*c*_ and *C*_*c*_ allows for the exact functionality of the impedance change to be determined, allowing for truly dynamic monitoring of the epithelial barrier disruption. For example, in the present case, a small but discernable increase in *R*_*c*_ (up to 475 kΩ) is evident after the addition of Cytochalasin B, and before the precipitous decrease. The cause for this increase is not fully understood, however the data may be explained by the fact that low concentrations of Cytochalasin B (nanomolar) have been demonstrated to give rise to increases in transepithelial resistance^29^,^30^, while larger doses may decrease TER^21^,^31^. Therefore, given the improved, unskewed measurement resolution, we may be observing an initial increase due to a perceived ‘lower dose’ followed by a decrease as the full amount of toxin diffuses to the cells. Such biphasic responses mean that being able to follow kinetics of cell responses to these stimuli, not limited by the measurement resolution, are very important to understand the mechanism of action.

The noise-based approach to monitoring cell properties is not only useful for attaining high resolution impedance traces, but also for monitoring the impedance of fast events in a continuous manner. In the specific case of barrier forming tissue monitoring, there are circumstances under which a rapid change in barrier properties due to drugs, toxins or electrical stimulation (electroporation) must be dynamically monitored. In the extreme case, the dynamics of cell death can be measured by recording the impedance of a confluent barrier-forming tissue layer.

**Figure 3:**
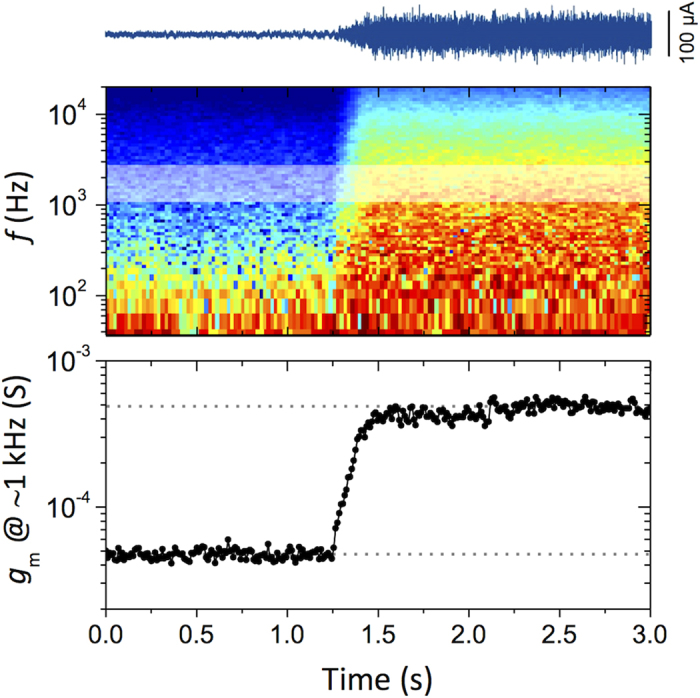
Short duration noise monitoring of MDCK I cell layer disruption with H_2_O_2_. Top: high pass filtered (1 Hz) drain current recording during addition of ~500 mM H_2_O_2_ to cultured MDCK I cells, Middle: Time-frequency analysis of the resulting transconductance (red: high, blue: low). Bottom: Magnitude of the transconductance at ~1 kHz. The resulting impedance change occurs over 250 μs, and is fully resolved by the measurement.

To demonstrate rapid changes in impedance, we monitor the death/disruption of cells upon addition of a high concentration of hydrogen peroxide (H_2_O_2_). Jimison and co-workers^24^ previously explored the dynamic disruption of Caco-2 cell barriers with H_2_O_2_ using gate voltage pulses, for a broad range of concentrations. Addition of 50 and 100 mM resulted in disruption faster than the 30 s spacing of applied voltage pulses. Using this information we chose to add a concentration that was sufficiently high to rapidly disrupt the cells, while monitoring such dynamics using noise. By monitoring the noise frequency content we are able to track the death of the epithelial cells via the disruption of the barrier properties of the cell monolayer (Fig. 3). The MDCK I barrier functions are shown to degrade at ~t = 1.25 s by observing a rapid increase in the *I*_*D*_ noise of the high-pass filtered (1 Hz) data. The frequency content of this current trace is visible via the time-frequency plot. From this data, we plot the transconductance at 1 kHz, alternatively, the cut off frequency could be quantified, or as above, the impedance (from *I*_*G*_ measurements). Regardless of the choice of data displayed, the functionality of the cell layer disruption occurs in 250 μs, and is fully resolved. It should be noted that over the short-term duration of the disruption, the DC transconductance remains unchanged (~2 mS), indicating that over this time frame, the device performance is not affected by the H_2_O_2_, and that the observed change is due to the disruption of the cell barrier layer.

The influence of prolonged noise on both cell layer and device were found to be negligible. Application of excessive gate voltage across a lipid bilayer can lead to bilayer rupture^32^,^33^, suggesting that 0 V-offset uniform white noise of 100 mV amplitude should cause minimal disruption to the biological system. Indeed, cell viability remains high when 100 mV uniform white noise is continuously applied for 4–5 hours (comparable to both frequency sweep and control experiments), see Supplemental Figure S5. Additionally, monitoring the frequency dependent transconductance before and after long-term bias shows little variation, suggesting minimal change in barrier tissue function, and device performance.

## Discussion

Noise-based monitoring brings high temporal resolution to impedance measurements of biological systems. A frequency sweep approach becomes inadequate in circumstances where the change in impedance occurs on the time scale of the measurement (in essence skewing the impedance spectra). In addition, the collected data lends itself to a broad array of established signal analysis tools. For example, depending on the frequency range of interest, more complex time-frequency analysis can be used, for example, a continuous wavelet transform. Many analysis routines can thus be borrowed from the fields of neuroscience and acoustics.

Using an applied gate bias of noise we have shown that the same transconductance and impedance spectra can be produced as with a harmonic frequency sweep. This dynamic monitoring can be utilized to track changes due to toxicological effects on cells, and is of high enough resolution to reliably record disruption events on the sub second time scale. The utilization of both noise and harmonic data (as in Fig. 2) allows for monitoring of both short time-scale toxicology and long term, chronic, effects on biological systems. The monitoring of rapid variations in cell monolayer properties is of importance for understanding the mechanism or pathway for cell or tissue disruption, as well as for quantification of dynamics.

Dynamic monitoring also has the advantage to gain additional information via multi-modal sensing. For example, combining a technique where the DC current yields useful data, while the high frequency spectral content provides impedance data is of interest both *in vivo* and *in vitro*. An enzymatic sensor, for example, can monitor low frequency changes due to H_2_O_2_ production by glucose oxidase in the presence of glucose, while simultaneously monitoring barrier tissue health or cell adhesion/confluence. Similarly, an electrode or transistor-based device can be used to monitor electrical activity (firing of neurons or cardiac cells) while monitoring the local impedance. In this case, the bands of activity need to be discernable and of high enough amplitude to separate the response of the environment to white noise and the signal from the electrogenic cells.

The utility of noise applies to both electrode and transistor recordings; the transistors in this case are able to amplify the low frequency signal and provide higher quality data. Combining both electrode-like and transistor recordings simultaneously has been shown to yield high quality data^20^. In this way, high resolution and dynamic monitoring of biological systems is possible – yielding data that provides richer and denser information that may prove critical for both fundamental and diagnostic bioelectronics applications.

## Methods

### Device Fabrication

Organic electrochemical transistors were fabricated as previously described^34^. Glass slides were cleaned by sonication in acetone, isopropyl alcohol, and DI water before a 2 min oxygen plasma treatment. Gold interconnects, conducting polymer, and an insulating Parylene-C layer were patterned photolithographically. Au contacts and interconnects were thermally evaporated and patterned via lift-off. Two layers of 2-μm Parylene-C were then deposited, separated by a layer of anti-adhesive. The active area is photolithographically defined, etched with O_2_ plasma, before spinning the CP formulation (Clevios PH1000, Heraeus Holding Gmbh, with 5 v/v% ethylene glycol, and 1 wt% (3-glycidyloxypropyl) trimethoxysilane). The top layer of Parylene-C serves as a sacrificial layer to simultaneously pattern the active area and insulating layer. Completed devices were rinsed, a glass well was affixed to the device array and soaked overnight in DI water. The wells were sterilized by soaking for 20 min in ethanol 70% in preparation for cell culture.

### OECT electrical characterization

All characterization was done with cell culture media as the electrolyte and a Ag/AgCl wire (Warner Instruments) as the gate electrode. The measurements were performed using a National Instruments PXIe-1062Q system. The channel of the OECT (*V*_*D*_) was biased using one channel of a source-measurement unit NI PXIe-4145. The gate voltage (*V*_*G*_) was applied and controlled using a NI PXI-6289 modular instrument. Before each experiment, the drain voltage is applied, and the device current is left to stabilize (typically < 2 min). For frequency-dependent characterization of the OECT, we used NI-PXI-4071 digital multimeters to measure simultaneously drain and gate current. The bandwidth measurements were performed by applying a sinusoidal modulation (∆*V*_*Gs*_ = 10 mV peak-to-peak, 1 Hz < *f* < 20 kHz), *V*_*D*_ as noted in the text, and measuring ∆*I*_*D*_ and ∆*I*_*G*_, and therefore *G*_*m*_ and |*Z*| as a function of frequency. The uniform white noise applied at the gate was generated using the NI PXI-6289 multifunction instrument, with various RMS amplitudes, and at a sampling rate of 100 kHz. All the equipment was controlled using customized LabVIEW software. Measurements were triggered through the built-in PXI architecture. The recorded signals were saved and analyzed using customized LabVIEW and MATLAB software. Time-frequency spectrograms, and impedance analysis/fitting (as in ref 20) were performed using custom MATLAB tools.

### Cell Culture

Madin-Darby canine kidney cells (MDCK I, HPACC (catalog no. 62106)) were cultured in DMEM low glucose media supplemented with 10% of fetal bovine serum, 430 mg/mL glutamine, 50 U/mL of penicillin, 50 μg/mL of streptomycin and 50 μg/mL of gentamicin. These products were purchased from Invitrogen. The cells were incubated in humidified atmosphere at 37 °C and 5% CO_2_. MDCK I was seeded at 5 × 10^4^ cell/cm^2^ in each well and growing for 3 days to obtain an epithelial monolayer. The media was replaced every 2 days. Cytochalasin B and H_2_O_2_ used for cell experiments were purchased from Sigma-Aldrich. A stock solution of 4.17 mM Cytochalasin B in dimethyl sulfoxide (DMSO, Fisher Scientific) was diluted in cell culture media before addition to the culture (final concentration: 8 μM). DMSO as a control was added in the same amount and in the same manner as the Cytochalasin B.

### Viability test

To observe the effect of the field or noise on the cells, we performed a viability test. After experimentation, MDCK I cells were washed with pre-warmed PBS 1X. Then a fresh solution of calcein-AM at 2 μg/mL (Sigma) and propidium iodide at 2 μg/mL (Sigma) was added to the cells. The fluorescence was observed after an incubation time at 37 °C using an inverted microscope Axio Observer Z1 (Carl Zeiss Microscopy).

## Additional Information

**How to cite this article**: Rivnay, J. *et al*. Using white noise to gate organic transistors for dynamic monitoring of cultured cell layers. *Sci. Rep*. **5**, 11613; doi: 10.1038/srep11613 (2015).

## Supplementary Material

Supplementary Information

## References

[b1] Alberts B. Molecular biology of the cell. (Garland Science, 2008).

[b2] Barberis A., Petrini E. M. & Mozrzymas J. W. Impact of Synaptic Neurotransmitter Concentration Time Course on the Kinetics and Pharmacological Modulation of Inhibitory Synaptic Currents. Frontiers in Cellular Neuroscience 5, 6 (2011). 21734864 10.3389/fncel.2011.00006 PMC3123770

[b3] Kholodenko B. N., Hancock J. F. & Kolch W. Signalling ballet in space and time. Nat. Rev. Mol. Cell Biol. 11, 414–426 (2010). 20495582 10.1038/nrm2901 PMC2977972

[b4] Kueh H. Y. & Mitchison T. J. Structural Plasticity in Actin and Tubulin Polymer Dynamics. Science 325, 960–963 (2009). 19696342 10.1126/science.1168823 PMC2864651

[b5] Spiering D. & Hodgson L. Dynamics of the Rho-family small GTPases in actin regulation and motility. Cell Adh. Migr. 5, 170–180 (2011). 21178402 10.4161/cam.5.2.14403 PMC3084983

[b6] Fernandez-Suarez M. & Ting A. Y. Fluorescent probes for super-resolution imaging in living cells. Nat. Rev. Mol. Cell Biol. 9, 929–943 (2008). 19002208 10.1038/nrm2531

[b7] Giaever I. & Keese C. R. Micromotion of mammalian cells measured electrically. Proc. Natl. Acad. Sci. USA. 88, 7896–7900 (1991). 1881923 10.1073/pnas.88.17.7896 PMC52411

[b8] Ramuz M. . Combined optical and electronic sensing of epithelial cells using planar organic transistors. Adv. Mater. 26, 7083–7090 (2014). 25179835 10.1002/adma.201401706 PMC4489338

[b9] Giaever I. & Keese C. R. Monitoring fibroblast behavior in tissue culture with an applied electric field. Proc. Natl. Acad. Sci. USA. 81, 3761–3764 (1984). 6587391 10.1073/pnas.81.12.3761 PMC345299

[b10] Giaever I. & Keese C. R. A morphological biosensor for mammalian cells. Nature 366, 591–592 (1993). 8255299 10.1038/366591a0

[b11] Lo C. M., Keese C. R. & Giaever I. Monitoring motion of confluent cells in tissue culture. Exp. Cell Res. 204, 102–109 (1993). 8416788 10.1006/excr.1993.1014

[b12] Lo C. M., Keese C. R. & Giaever I. Impedance analysis of MDCK cells measured by electric cell-substrate impedance sensing. Biophys. J. 69, 2800–2807 (1995). 8599686 10.1016/S0006-3495(95)80153-0 PMC1236517

[b13] Wegener J., Keese C. R. & Giaever I. Electric cell-substrate impedance sensing (ECIS) as a noninvasive means to monitor the kinetics of cell spreading to artificial surfaces. Exp. Cell Res. 259, 158–166 (2000). 10942588 10.1006/excr.2000.4919

[b14] Keese C. R., Wegener J., Walker S. R. & Giaever I. Electrical wound-healing assay for cells *in vitro*. Proc. Natl. Acad. Sci. USA. 101, 1554–1559 (2004). 14747654 10.1073/pnas.0307588100 PMC341773

[b15] Wegener J., Abrams D., Willenbrink W., Galla H. J. & Janshoff A. Automated multi-well device to measure transepithelial electrical resistances under physiological conditions. BioTechniques 37, 590, 592–594, 596-597 (2004). 10.2144/04374ST03 15517971

[b16] Voelker M. & Fromherz P. Nyquist Noise of Cell Adhesion Detected in a Neuron-Silicon Transistor. Phys. Rev. Lett. 96, 228102 (2006). 16803347 10.1103/PhysRevLett.96.228102

[b17] Rivnay J., Owens R. M. & Malliaras G. G. The Rise of Organic Bioelectronics. Chem. Mater. 26, 679 (2014).

[b18] Strakosas X., Bongo M. & Owens R. M. The organic electrochemical transistor for biological applications. J. Appl. Polym. Sci., 132, 41735 (2015).

[b19] Khodagholy D. . High transconductance organic electrochemical transistors. Nat. Commun. 4, 2133 (2013). 23851620 10.1038/ncomms3133 PMC3717497

[b20] Rivnay J. . Organic electrochemical transistors for cell-based impedance sensing. Appl. Phys. Lett. 106, 043301 (2015).

[b21] Stevenson B. R. & Begg D. A. Concentration-dependent effects of cytochalasin D on tight junctions and actin filaments in MDCK epithelial cells. J. Cell Sci. 107, 367–375 (1994). 8006058 10.1242/jcs.107.3.367

[b22] Shen L., Weber C. R. & Turner J. R. The tight junction protein complex undergoes rapid and continuous molecular remodeling at steady state. J. Cell Biol. 181, 683–695 (2008). 18474622 10.1083/jcb.200711165 PMC2386107

[b23] Rivnay J. . Organic Electrochemical Transistors with Maximum Transconductance at Zero Gate Bias. Adv. Mater. 25, 7010–7014 (2013). 24123258 10.1002/adma.201303080

[b24] Jimison L. H. . Measurement of Barrier Tissue Integrity with an Organic Electrochemical Transistor. Adv. Mater. 24, 5919–5923 (2012). 22949380 10.1002/adma.201202612

[b25] Tria S. A. . Dynamic monitoring of Salmonella typhimurium infection of polarized epithelia using organic transistors. Adv. Healthc. Mater. 3, 1053–1060 (2014). 24497469 10.1002/adhm.201300632

[b26] Goddette D. W. & Frieden C. The kinetics of cytochalasin D binding to monomeric actin. J. Biol. Chem. 261, 15970–15973 (1986). 3782101

[b27] Yamada S., Pokutta S., Drees F., Weis W. I. & Nelson W. J. Deconstructing the cadherin-catenin-actin complex. Cell 123, 889–901 (2005). 16325582 10.1016/j.cell.2005.09.020 PMC3368712

[b28] Faria G. C. . Organic Electrochemical Transistors as Impedance Biosensors. MRS Commun. 4, 189 (2014).

[b29] Nybom P. & Magnusson K. E. Modulation of the junctional integrity by low or high concentrations of cytochalasin B and dihydrocytochalasin B is associated with distinct changes in F-actin and ZO-1. Biosci. Rep. 16, 313–326 (1996). 8896790 10.1007/BF01855015

[b30] Van Itallie C. M., Fanning A. S., Bridges A. & Anderson J. M. ZO-1 Stabilizes the Tight Junction Solute Barrier through Coupling to the Perijunctional Cytoskeleton. Mol. Biol. Cell 20, 3930–3940 (2009). 19605556 10.1091/mbc.E09-04-0320 PMC2735491

[b31] Shen L. & Turner J. R. Actin depolymerization disrupts tight junctions via caveolae-mediated endocytosis. Mol. Biol. Cell 16, 3919–3936 (2005). 15958494 10.1091/mbc.E04-12-1089 PMC1196308

[b32] Bernards D. A., Malliaras G. G., Toombes G. E. S. & Gruner S. M. Gating of an organic transistor through a bilayer lipid membrane with ion channels. Appl. Phys. Lett. 89, 05305 (2006).

[b33] Ghosh P. M., Keese C. R. & Giaever I. Monitoring electropermeabilization in the plasma membrane of adherent mammalian cells. Biophys. J. 64, 1602–1609 (1993). 8324195 10.1016/S0006-3495(93)81531-5 PMC1262488

[b34] Khodagholy D. . *In vivo* recordings of brain activity using organic transistors. Nat. Commun. 4, 1575 (2013). 23481383 10.1038/ncomms2573 PMC3615373

