# Supplementary material for: Using white noise to gate organic transistors for dynamic monitoring of cultured cell layers
Source: Sci Rep. 2015 Jun 26;5:11613. doi: 10.1038/srep11613 (PMC4481393; doi:10.1038/srep11613)
Supplement: Supplementary Information [file srep11613-s1.pdf]

Supplementary Information

Using white noise to gate organic transistors for dynamic monitoring of cultured cell layers

Jonathan Rivnay[1], Pierre Leleux[1,2], Adel Hama[1], Marc Ramuz[1], Miriam Huerta[1], George G. Malliaras[1], Roisin M. Owens[1]

[1]*Deptartment of Bioelectronics, Ecole Nationale Superieure des Mines, CMP-EMSE, MOC, 13541 Gardanne, France*

[2]*Microvitae Technologies, Hôtel Technologique, Route de Valbrillant, 13590 Meyreuil, France*

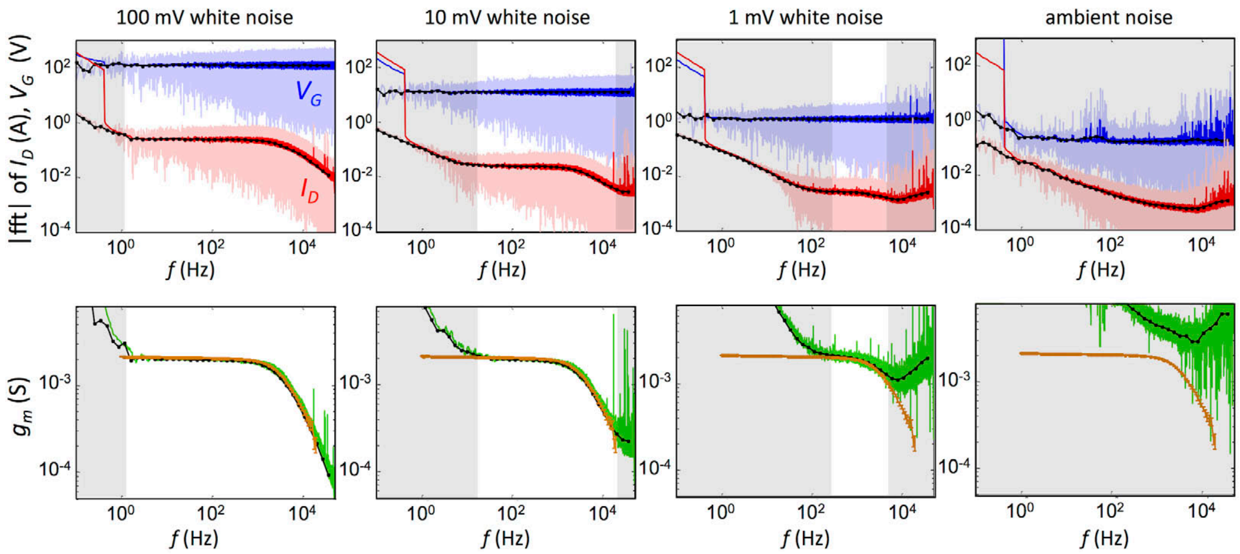

Supplementary Figure S1. Influence of noise amplitude. Top: FFT of measured gate voltage (blue) and drain current (red) for applied uniform white noise of 100 mV, 10 mV, 1 mV, and for reference, ambient (environmental) noise. Bottom: resulting frequency-dependent transconductance [|FFT(I_D)|/|FFT(V_G)|]. The orange trace is the frequency dependent transconductance from harmonic measurements. Light colors are the raw FFTs, darker colors are the smoothed FFTs (50 pts), and black lines are the FFT data smoothed on a log scale. Shaded grey areas are the frequency ranges where the ambient noise dominates the signal.

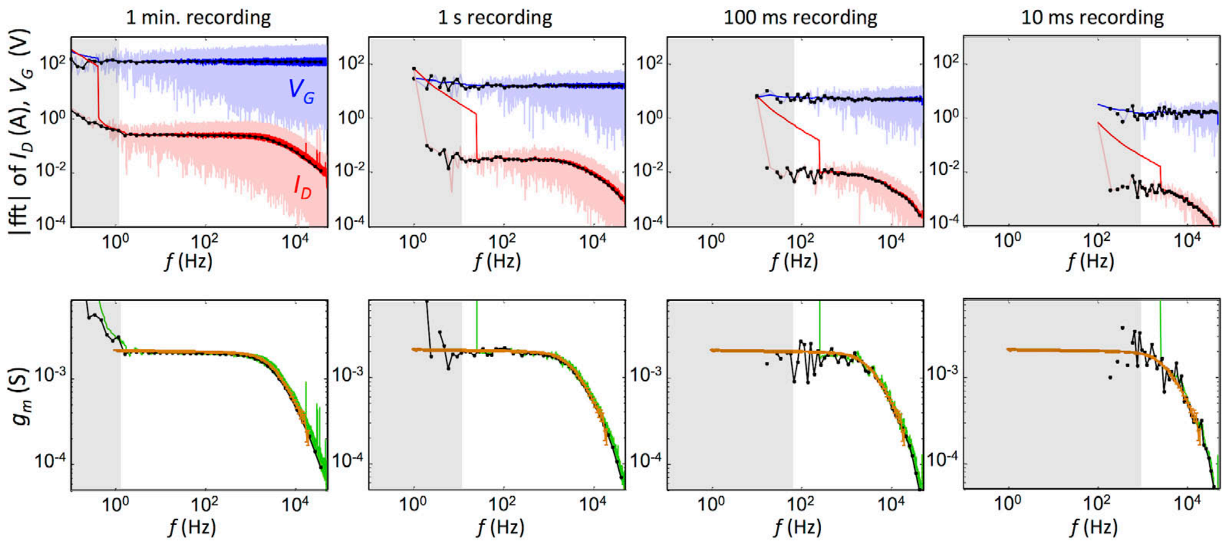

Supplementary Figure S2. Influence of noise duration. Top: FFT of measured gate voltage (blue) and drain current (red) for 100 mV uniform white noise applied for 1 min., 1 s, 100 ms, and 10 ms. Bottom: resulting frequency-dependent transconductance [$|FFT(I_D)|/|FFT(V_G)|$]. The orange trace is the frequency dependent transconductance from harmonic measurements. Light colors are the raw FFTs, darker colors are the smoothed FFTs (50 pts), and black lines are the FFT data smoothed on a log scale. Shaded grey areas are the frequency ranges where short duration recording limits low frequency transconductance determination.

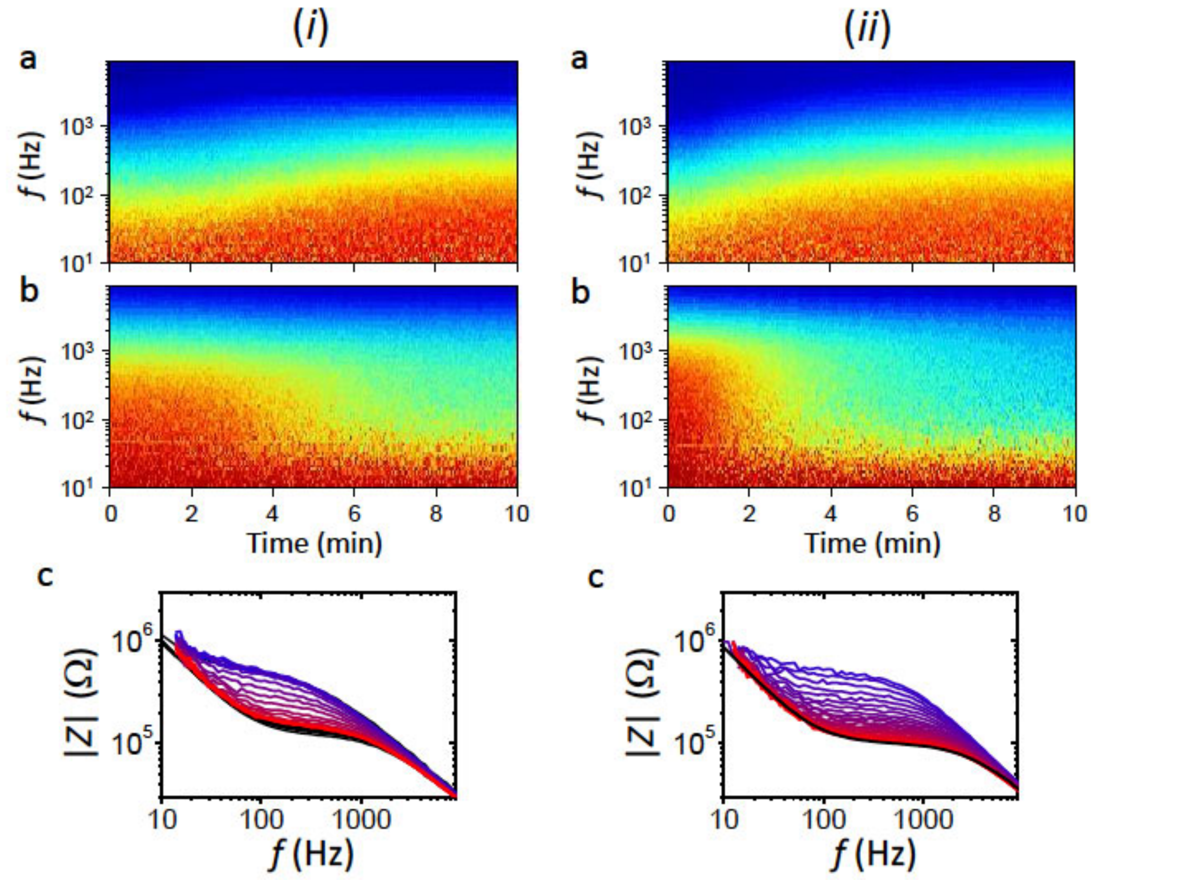

Supplementary Figure S3. Cyt B noise experiment reproducibility. Two repetitions of the Cyt B noise experiment (*i*), (*ii*), with time frequency spectrogram of transconductance (a), and impedance (b) for MDCK I cell layers cultured on top of an OECT on addition of Cyt B (as in main text Figure 2), (red: high, blue: low). The Cyt B is added, in this figure, at t=0 min. (c) Impedance snapshots during the noise experiment, with blue indicating the early noise experiment time points, and red the later time points. Black traces are from the harmonic frequency sweep impedance data. The approximate time course and extracted impedance fitting values are comparable between the two experiments. For experiment (*i*): C_{OECT} = 14.9 nF, R_c varies from 430 kΩ to 120 kΩ, and C_c from 4.5 nF to 4.9 nF from t=0 min to t=14 min. For experiment (*ii*): C_{OECT} = 18.6 nF, R_c varies from 490 kΩ to 80 kΩ, and C_c from 3.1 to 3.9 nF from t=0 min to t=14 min.

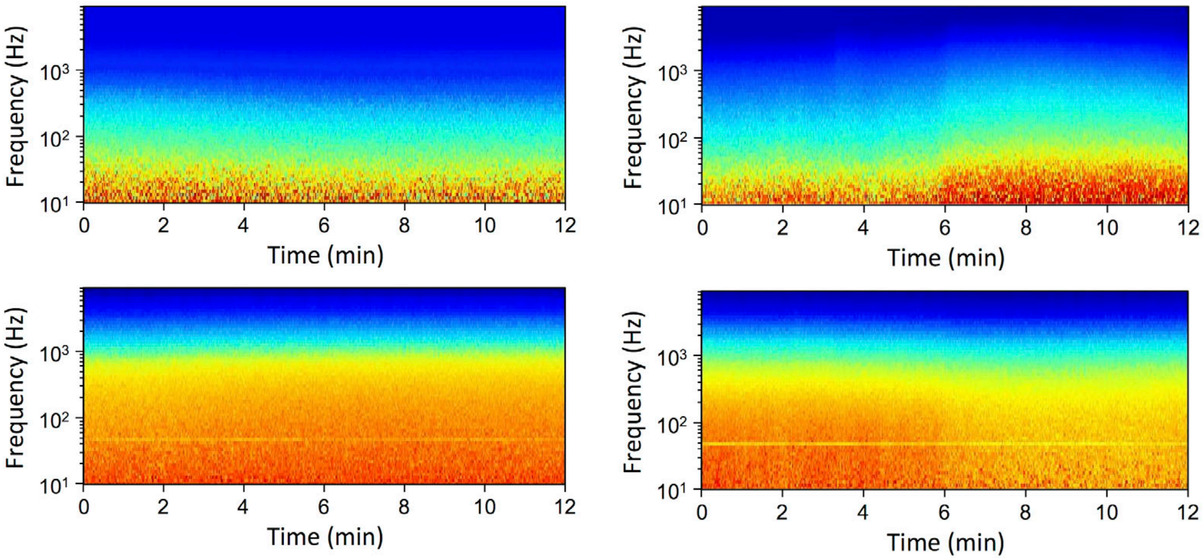

Supplementary Figure S4. Continuous noise experimental controls. Time frequency spectrogram of transconductance (top), and impedance ($|Z|$) for MDCK I cell layers cultured on top of an OECT on addition of 10 µL of cell culture medium (left) and 10 µL of DMSO at time t=2.5 min. Red is high, Blue is low.

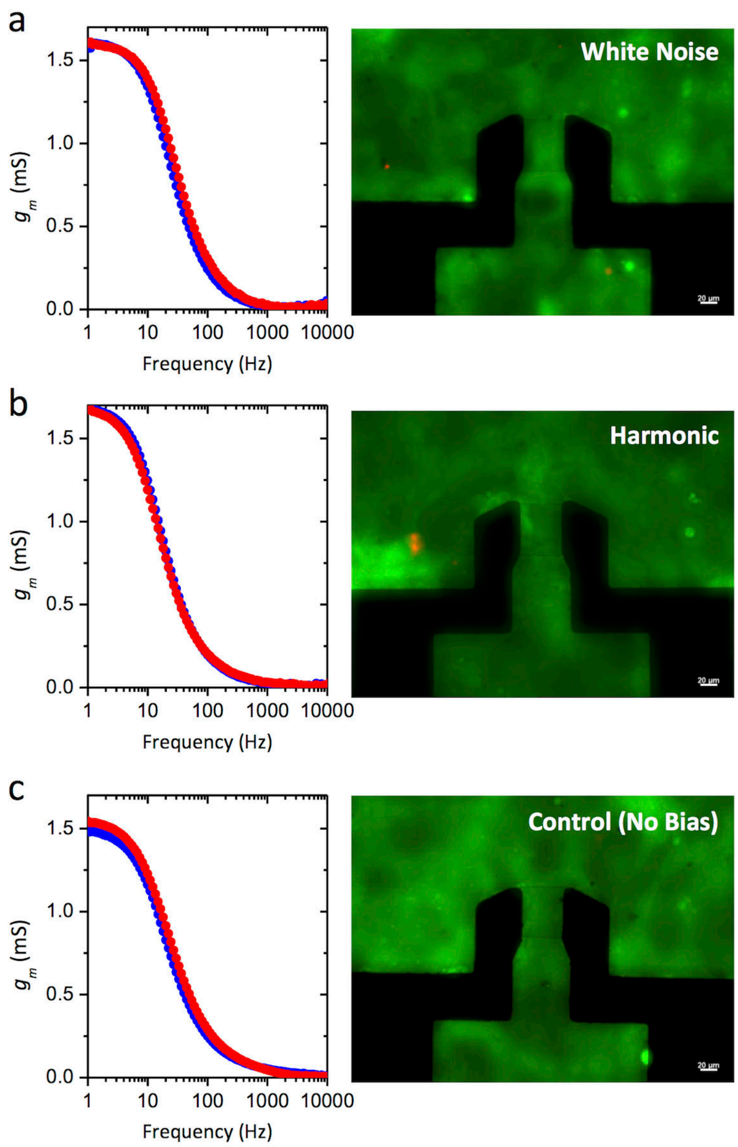

Supplementary Figure S5. Cell layer and device response to long-term noise sourcing. Left: Frequency-dependent (harmonic) transconductance performed before (blue) and after (red) the long term biasing in a controlled incubator environment to show maintenance of cell-barrier function and device performance. Maintaining low frequency transconductance indicates that the transistor is unaffected, while the unchanged cut-off frequency shows that the cell layer retains its barrier function. Right: Viability assay (Calcein AM, Propidium Iodide) on MDCK I cell layer cultured on a 50x50 μm^2 OECT (Green: live cells, red: dead cells, black areas are the contact lines for the OECT). Viability assay is performed after 5 hours of gate voltage application of 100 mV white noise (**a.**), 10 mV amplitude harmonic frequency sweeps (**b.**), and a control device, where no gate bias is applied for the same duration, stored in the same conditions (**c.**). V_D in all cases is -0.3 V.
